# Opportunities for Post-Graduate Study

**Published:** 1910-09-03

**Authors:** 


### Post-Graduate Medical Section.

### OPPORTUNITIES FOR POST-GRADUATE STUDY.

BY A GRADUATE STUDENT.

The registered practitioner who desires to undertake reseach work, no matter in what form, will find his resolve fully justified by the experience which post-registration work, earnestly and seriously carried out, brings. The first question is the choice of a speciality, if he wishes to specialise, or a decision between the allurements of specialism and those of general study. This is a question which each practitioner must decide for himself. Natural bent and individual inclination will determine the choice. When a speciality is chosen, be it the eye, ear, throat, heart, stomach, or any one of the numerous departments in which modern medicine has been divided, the next question will be that of ways and means. There is a prevailing notion, utterly erroneous, that graduate study is very expensive, and that no one who has not a well-filled purse can afford to visit various schools and undertake special work in special institutions or classes. As a matter of fact, a year of graduate study, pursued at a Continental centre, need not cost the student more than a year of ante-registration work at a London hospital. Travelling expenses are heavy, it is true, especially when the selected centre is far from the base, as Vienna, Breslau, or Königsberg, but, on the other hand, the living expenses are comparatively slight, while class fees are low in proportion to those demanded by London hospitals. If the student has no ambition to visit foreign countries and observe foreign methods, he can achieve much in this country. The graduate study institutions in London, for instance, are exceptionally good. One has to go far afield to find finer clinical instruction than is given at the Seamen's (Dreadnought) Hospital, at the West London, or at the Tottenham Hospital, while the Polyclinic in Chenies Street has a well-deserved reputation as a graduate school. In addition, there are numerous special classes given by surgeons and physicians at various hospitals in the Metropolis and in the provinces, which allow of a wide choice and discrimination. They are far from being equally good, and the student would do well to consider seriously in each case the peculiar merits of the class he designs to join or of the course which he wishes to take. Some of these classes at the general hospitals are given by men who have had absolutely no experience in graduate teaching. They may be admirable—as they doubtless are—for students preparing for examination, but from a graduate student's point of view they are worthless. The practitioner desires teaching such as he cannot get in books. Such teaching can only be the outcome of experience, and no man who has not had a comparatively wide clinical experience ought to offer himself as a graduate teacher. The fact is very often overlooked, especially by young assistant physicians and surgeons who offer to give clinical instruction to post-graduate men. The student who wishes clinical instruction, therefore, should join a class which is presided over by a senior member of the staff, one who has had much experience, and who has an established reputation. On the other hand, for theoretical subjects, and for examination work, a younger teacher is preferable. He has the latest methods at his fingers' ends, he is primed with novelties, and his knowledge of examination work is comparatively fresh. So, also, if the student wishes to learn how to handle a new instrument or to undertake special research work which does not demand ward attendance, he will find the young teacher a help. Graduate students for all practical purposes may be said to fall into two categories—those who study with the object of passing higher examinations, and those who are imbued with the real love for knowledge, and pursue it with an enthusiastic desire to broaden their outlook. A middle class exists in which these two aims are partly combined. It is obvious that the demands of the first class will not be exactly those of the second. The former needs syllabus work, which is essentially hack-work in which the element of cram is conspicuous. The latter needs experience and experiment, practical work, not along stereotyped lines nor devised to meet the requirements of an examination, but generalised without being vague—everything, in short, that tends to make a "full man" in the best sense of the term.

Having made up his mind as to his needs, the student who wishes to do work at home, and who has not the time, means, or inclination to go abroad, cannot do better than write to or interview one of the deans of the graduate schools in London or the provinces. We possess, unfortunately, no central controlling body such as exists in Germany, where the inquirer can get information regarding teachers and courses. He must therefore make his own selection, and it may be said that he will find the authorities most courteous and willing to assist him in the choice of a school or class. If he does not already know the teacher, a glance at the Directory will supply him with valuable hints, but a much better plan is to attend one of the lectures or demonstrations and judge teacher and fellow-students for himself. And here let it be added that when the course or class is one to which unregistered practitioners—*i.e.* students—are allowed, the post-graduate will do well to shun it, for the teaching is not of the kind he requires, unless it happens to be a special class for one of the higher preliminary examinations. The teacher will be found ready and willing to devote himself to the student in the real graduate study course, and will do his utmost to supply the latter with practical work. A preliminary interview will settle the question of time, fees, instruments and books, and usually there is a marked tendency to make the path of the student as smooth as possible, to fall in with his wishes and to meet him half-way.

The true post-graduate student, he of the second class, will not be content with home experience merely. He will wander abroad, not, like Mr. Tarboys in Charles Reade's charming romance, "with a mind encased in non-conductors," but systematic, although critical. He learns to appreciate on a large scale—a faculty someone calls genius somewhere—and appreciating to admire. Above all he acquires experience, and teaches himself by the mere matter of observation. If he wishes to do special work, he will usually find plenty of opportunities; but a necessary preliminary to successful work at a foreign clinic is an acquaintance with the language which is spoken in it. The student's plan of operation when in quest of study abroad must vary with the country or the centre to which he proceeds. If it is his intention to go to Germany or Austria (or for that matter any Continental centre), the best advice that can be given him is, "Sit down and write a letter—in English if you like and do not know sufficient German to appreciate the distinction between *Wohlgeboren* and *Hochwohlgeboren*—to the Director of the Kaiserin Frederich Haus, Luisenplatz, Berlin, explaining in it clearly what you wish to do, the time at your disposal, and the centre or centres you desire to visit, and enclosing with it an addressed envelope for a reply." This will ensure you a courteous letter in return from Professor Kutner, who knows everything about graduate study everywhere, giving you all the information you require, and sending you any prospectus or literature that may be useful. If possible go to Berlin and see the Professor, see the magnificent institution he presides over and which has no counterpart anywhere else, and find out, as you can easily do, the nature of graduate study abroad by attending a lecture of Oppenheim, Krause, or Klapp. Similarly, if your inclinations prompt you to visit America, where graduate study has of late years made astonishing strides, communicate with the New York Post-Graduate College, Second Avenue and Twentieth Street, New York. The courteous superintendent, Dr. Frederic Brush, is always willing to give information to genuine graduate students, and he is fully acquainted with all the opportunities for graduate study on the other side of the Atlantic. His own school is one of the finest and most elaborately equipped in the world, and his ideal of graduate study is one that appeals to every true graduate student.

Having fixed on his centre, the student will lose no time in taking up his work in earnest. Patience and courtesy, toleration and good temper will carry him very far. He will have many difficulties to encounter and overcome, and doubtless appreciation of foreign methods will at first be elbowed out of his mind by an obstinate preference for more familiar routine work. It is difficult to be a genius! But in time he will learn to appreciate, to discriminate, and to be just in his criticism, instead of insular and prejudiced.

A word in conclusion as to the cost. From time to time articles have appeared in The Hospital giving detailed accounts of the opportunities for graduate study at various Continental centres, with details of cost. The writer has recently attempted to arrive at some conclusion with regard to the question of expense by taking the expenses of several graduate students from different centres and striking an average. In these accounts only the pure expenses of living, travelling, and special courses were taken. The individual sums differed somewhat widely, although the students selected were men who were enthusiasts and who were to a certain extent economical. Out of twelve such accounts taken at random the average worked out to 8s. 6d. per day for German and 11s. per day for French centres. This is probably a high average. A graduate student should be able to live comfortably and get all the work he can find the time to do at any Continental centre at a cost of £10 per month, this of course including special classes.

